# Impulsivity and Sensation Seeking in Bipolar Disorder: Association with Impulse Control Disorders

**DOI:** 10.1192/j.eurpsy.2026.11193

**Published:** 2026-07-31

**Authors:** F. Sahnoun, S. Ellouze, Y. Kammoun, S. Frikha, N. Halouani, M. Turki, J. Aloulou

**Affiliations:** Psychiatry “B” Department, Hédi Chaker University Hospital, Sfax, Tunisia

## Abstract

**Introduction:**

Bipolar disorder is often characterized by heightened impulsivity and sensation seeking, which may reflect a latent vulnerability to, or the coexistence of, Impulse Control Disorders (ICDs). Identifying the role of these traits may improve early detection and management of ICDs in this population.

**Objectives:**

To examine the relationship between impulsivity, sensation seeking, and ICDs among bipolar patients.

**Methods:**

This cross-sectional descriptive and analytical study included patients with bipolar disorder, during euthymia phase and receiving psychiatric follow-up in the Psychiatry “B” Department of Hédi Chaker University Hospital of Sfax, Tunisia.

Bipolar patients were assessed using the Barratt Impulsiveness Scale in its arabic dialect version (BIS-11) and the Sensation Seeking Scale of Zuckerman (SSS). ICDs were identified using the modified Minnesota Impulsive Disorders Interview (mMIDI).

**Results:**

A total of 72 patients with bipolar disorder participated in our study. Their mean age was 44.13 years, and the sex ratio (F/H) was 1.88. The mean duration of illness was 17.04 years. Most patients (83.3%) had bipolar I disorder. Overall, 33.3% of participants presented at least one impulse control disorder (ICD).

The mean BIS-11 score among patients was 57.07±11,18, and a marked level of impulsivity was observed in 12.5% of cases. The mean Sensation Seeking Scale (SSS) score was 9.68 ± 5.79.

Impulse control disorders were significantly associated with the total BIS-11 score (p < 0.001), the total SSS score (p = 0.004), as well as with the SSS subscales of thrill and adventure seeking (p = 0.046), disinhibition (p = 0.022), and boredom susceptibility (p = 0.028).

**Conclusions:**

The strong association between impulsivity, sensation seeking, and ICDs in bipolar disorder suggests that these personality dimensions play a central role in the expression of impulsive behaviors. Integrating psychological assessment and specific psychotherapeutic strategies could enhance the overall management of bipolar patients.

**Disclosure of Interest:**

None Declared

